# Supplementary material for: Cancer Cell Biomechanical Properties Accompany Tspan8-Dependent Cutaneous Melanoma Invasion
Source: Cancers (Basel). 2024 Feb 6;16(4):694. doi: 10.3390/cancers16040694 (PMC10887418; doi:10.3390/cancers16040694)
Supplement: Supplementary file 1 [file cancers-16-00694-s001.zip › cancers-2804031-supplementary figures.pdf]

Supplementary Figures

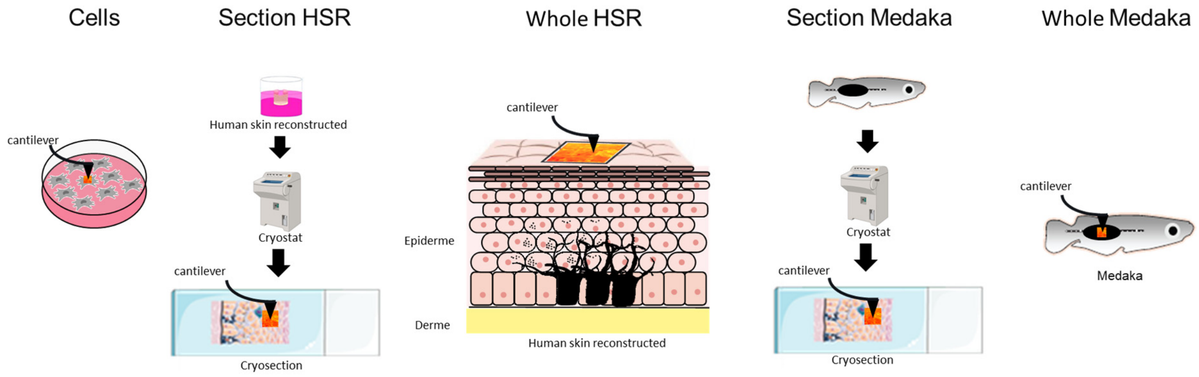

Supplementary Figure S1. Schematics of stiffness measurement in cultured-cells, sections from HSR, whole HSR, sections from Medaka skin and whole Medaka melanoma.

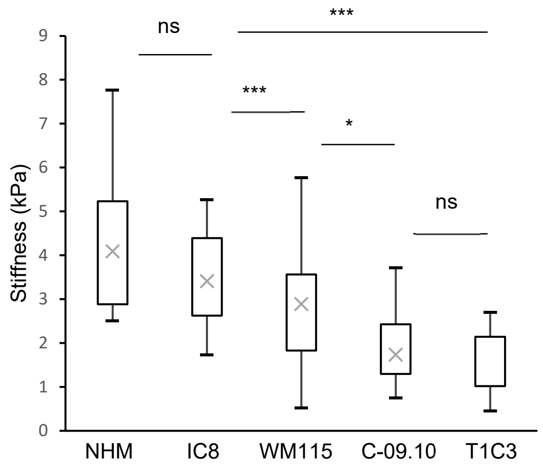

Supplementary Figure S2. Global measurement of melanoma cell stiffness during tumor progression with Normal Human Melanocytes (NHM), which corresponds to primary melanocytes, or IC8, WM115, C-09.10 and T1C3, which are non-invasive melanoma cells to highly invasive melanoma cells in the dermis respectively. * $p < 0.05$; *** $p < 0.001$; ns non-significant.
