# Supplementary figures and images for: Cancer Cell Biomechanical Properties Accompany Tspan8-Dependent Cutaneous Melanoma Invasion
Source: Cancers (Basel). 2024 Feb 6;16(4):694. doi: 10.3390/cancers16040694 (PMC10887418; doi:10.3390/cancers16040694)

Figure 2c

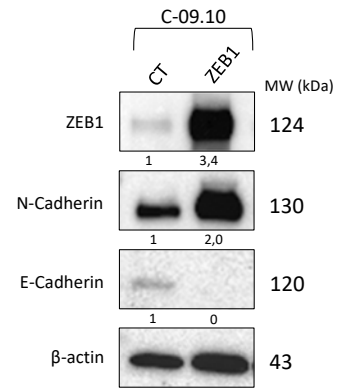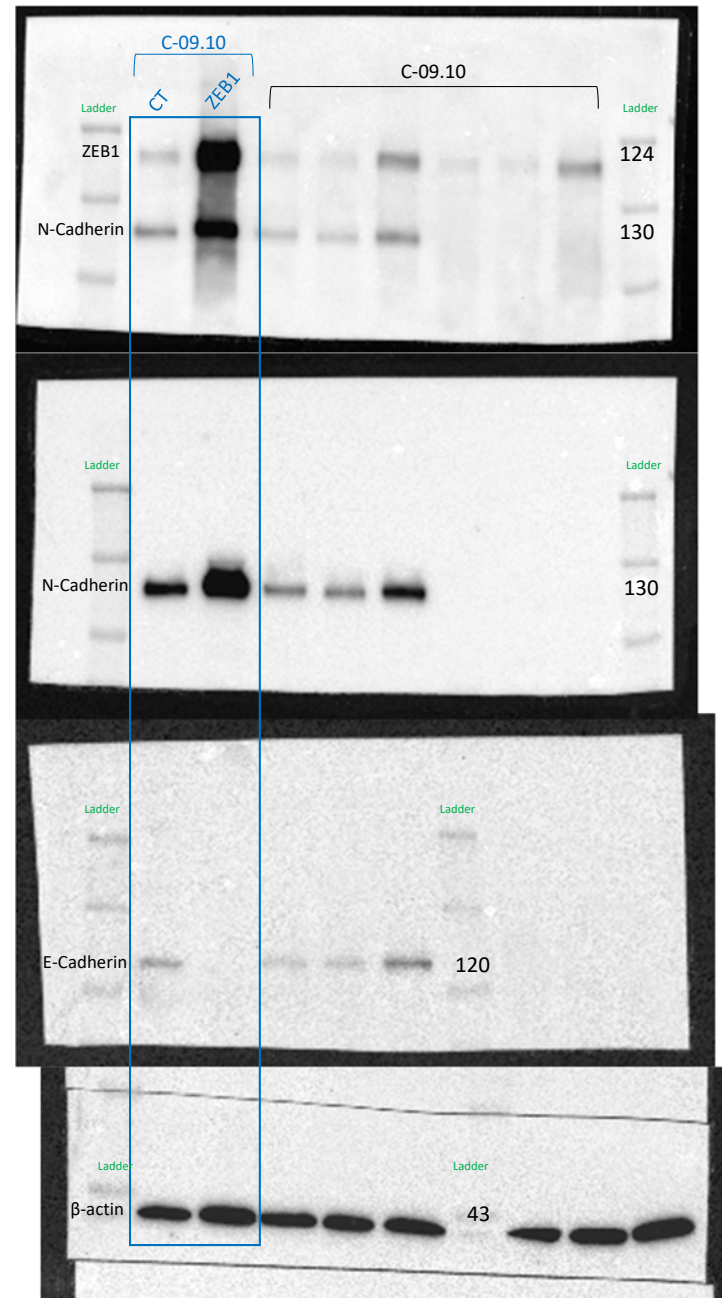

Figure 2e

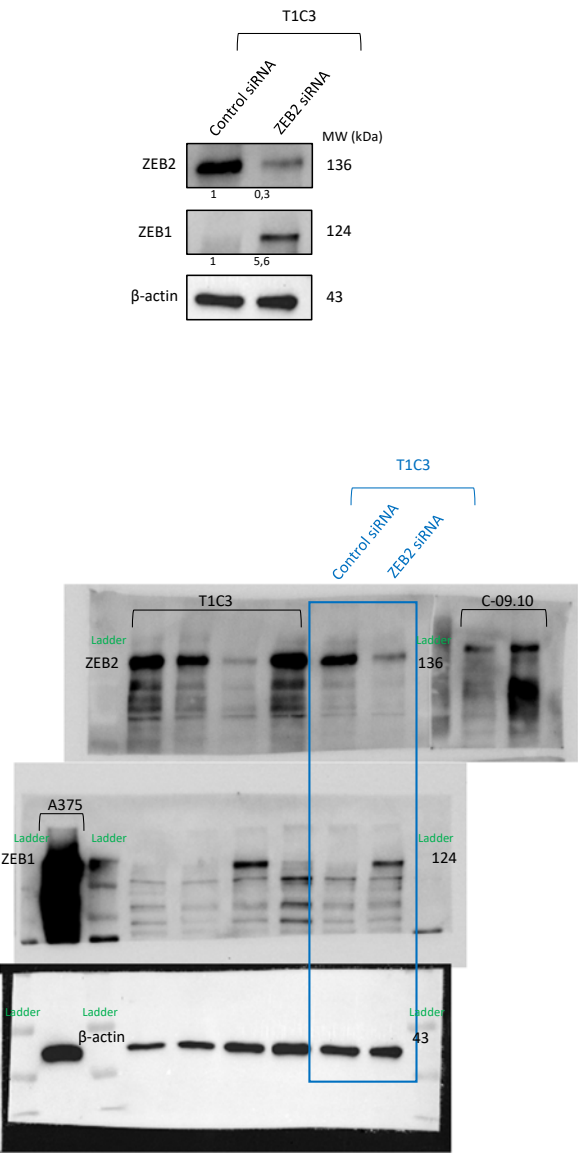

Figure 2e

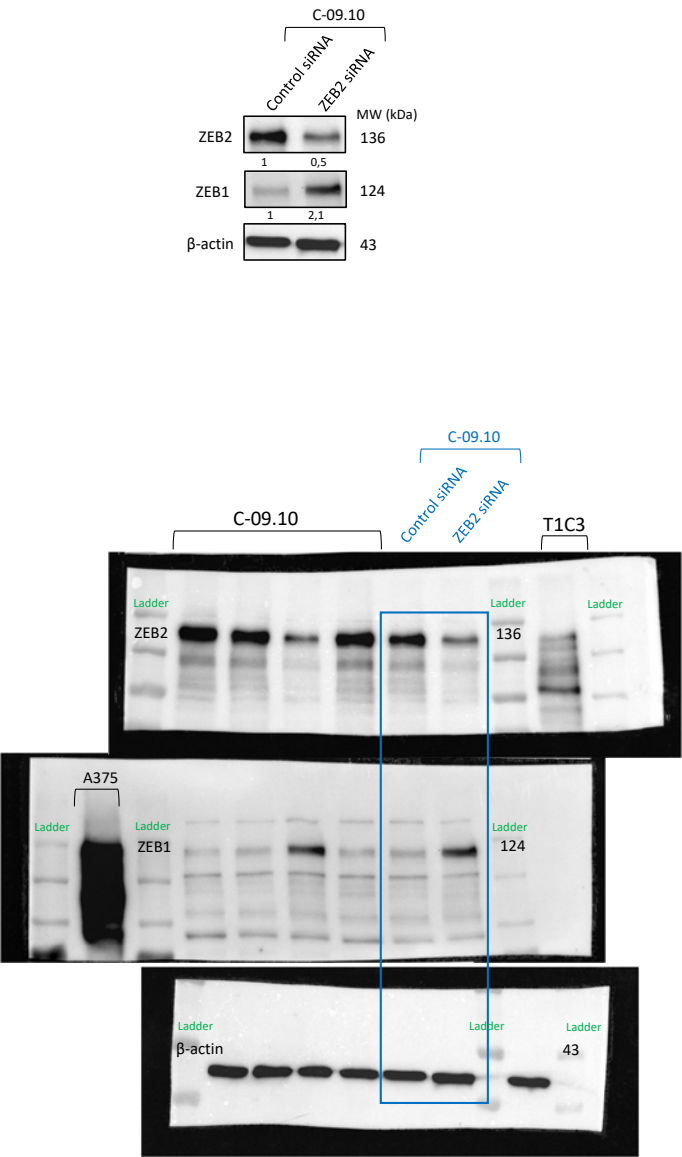

Figure 3b

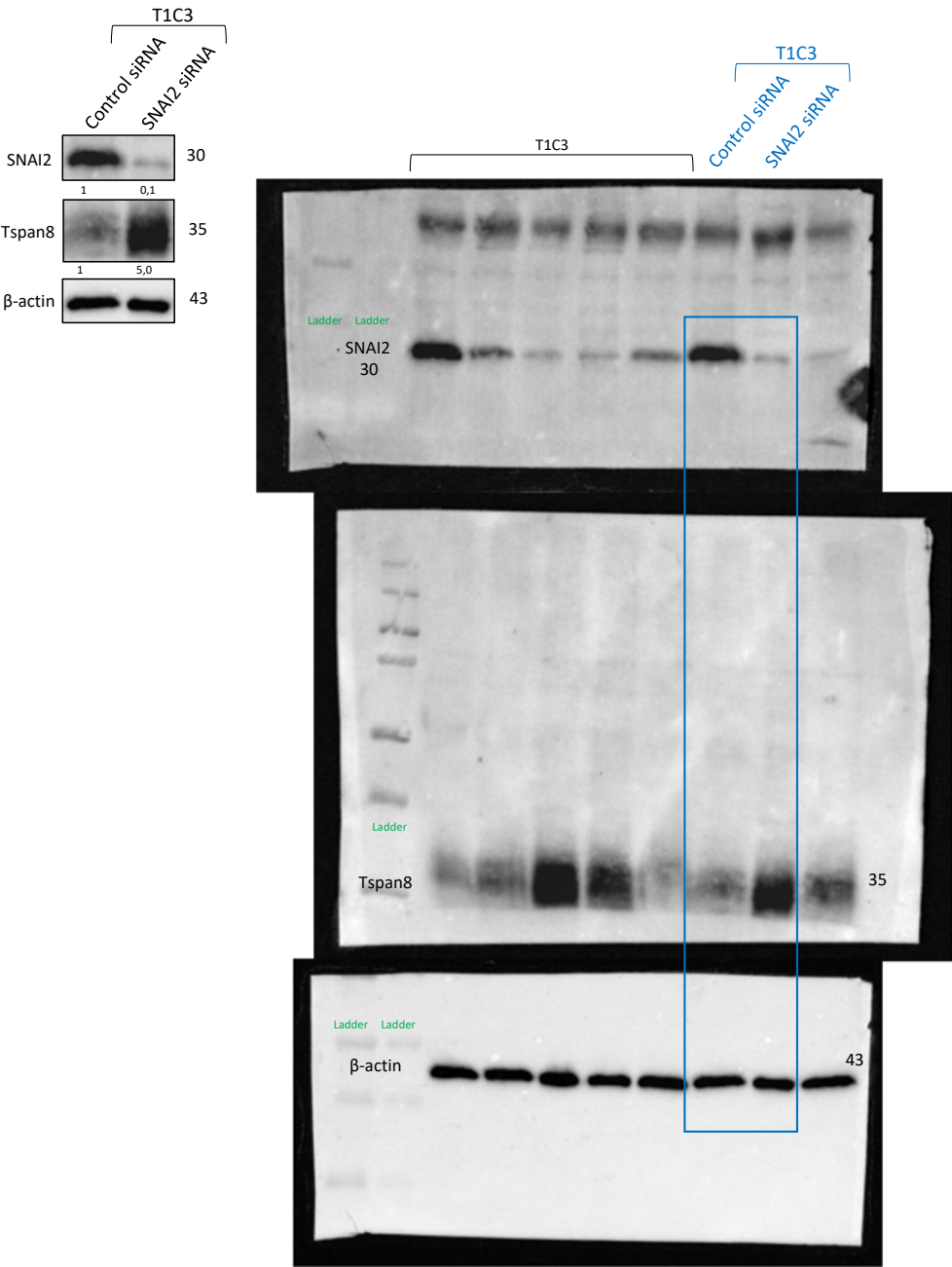

Figure 3b

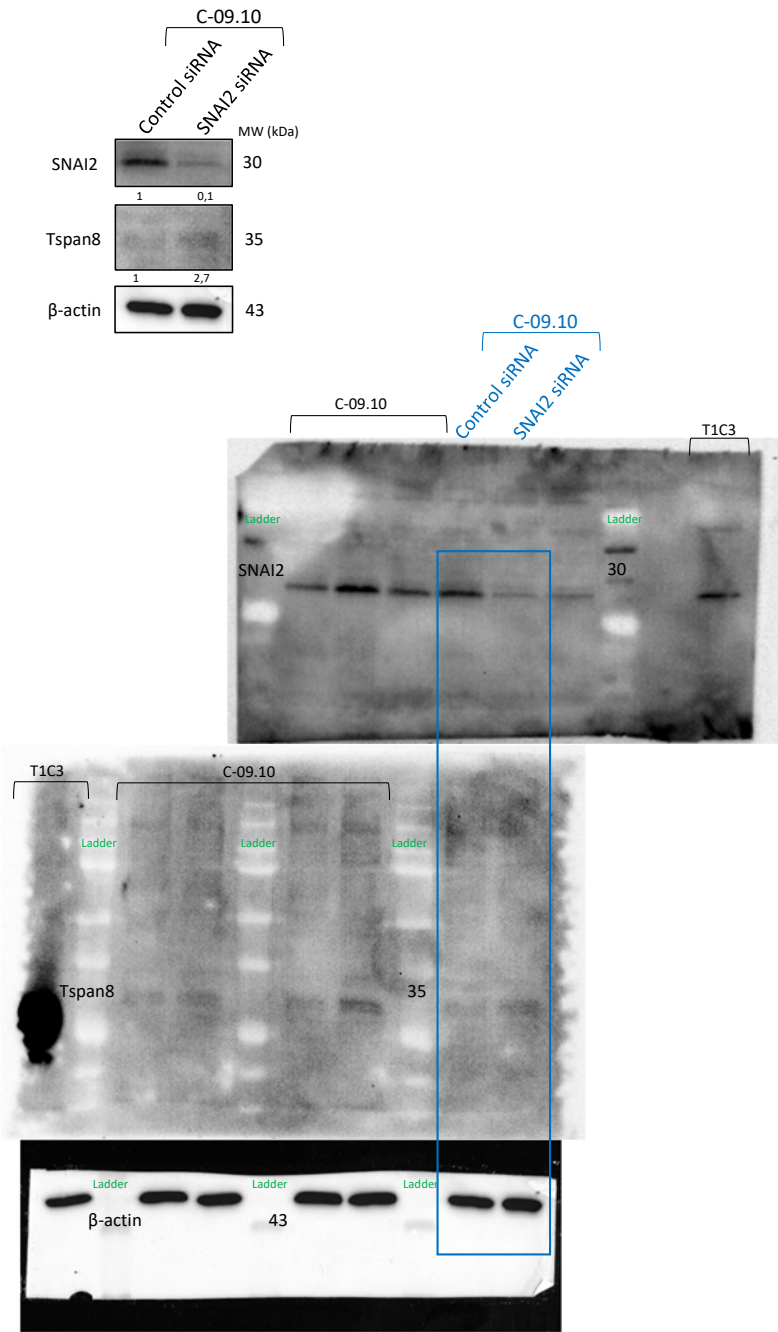

Figure 3b

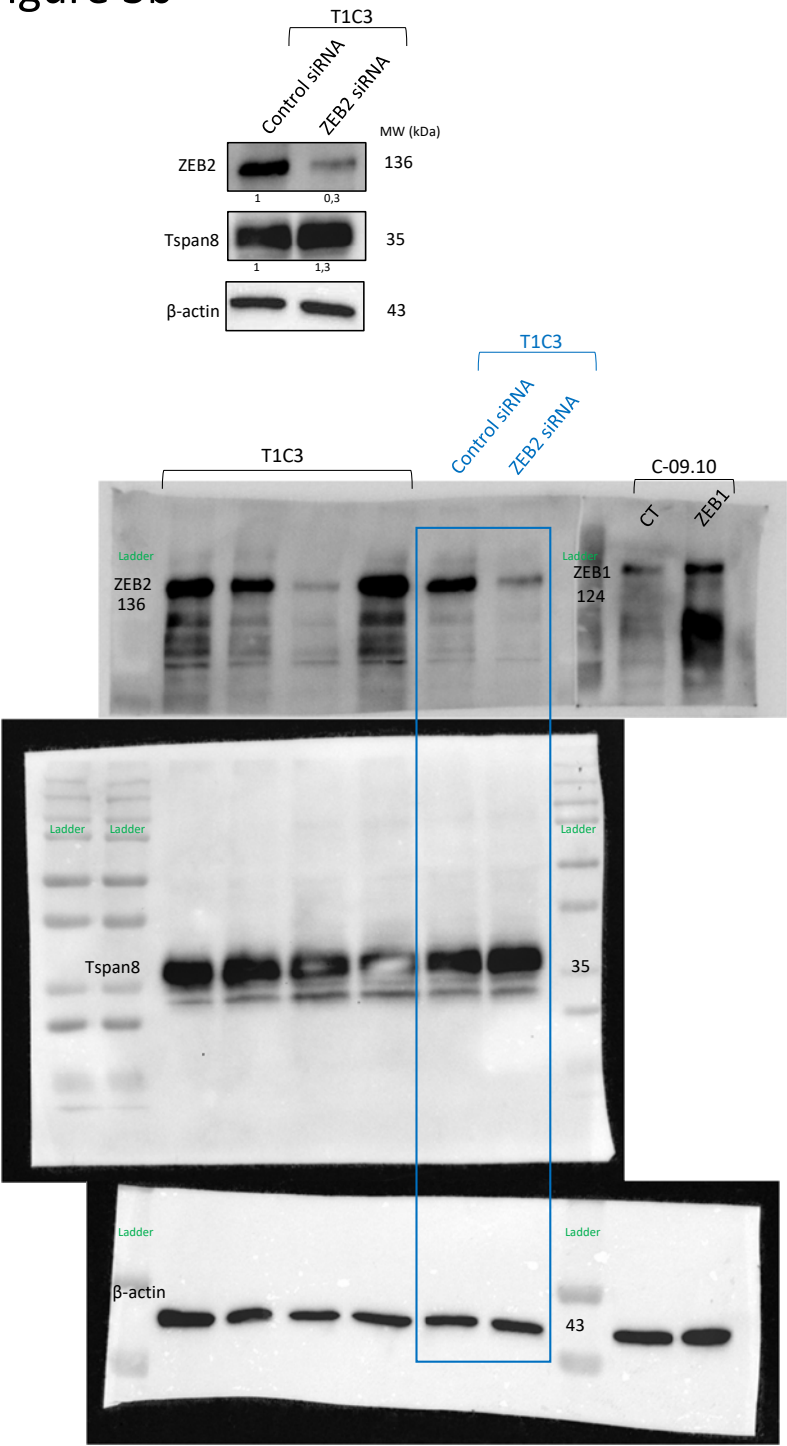

Figure 3b

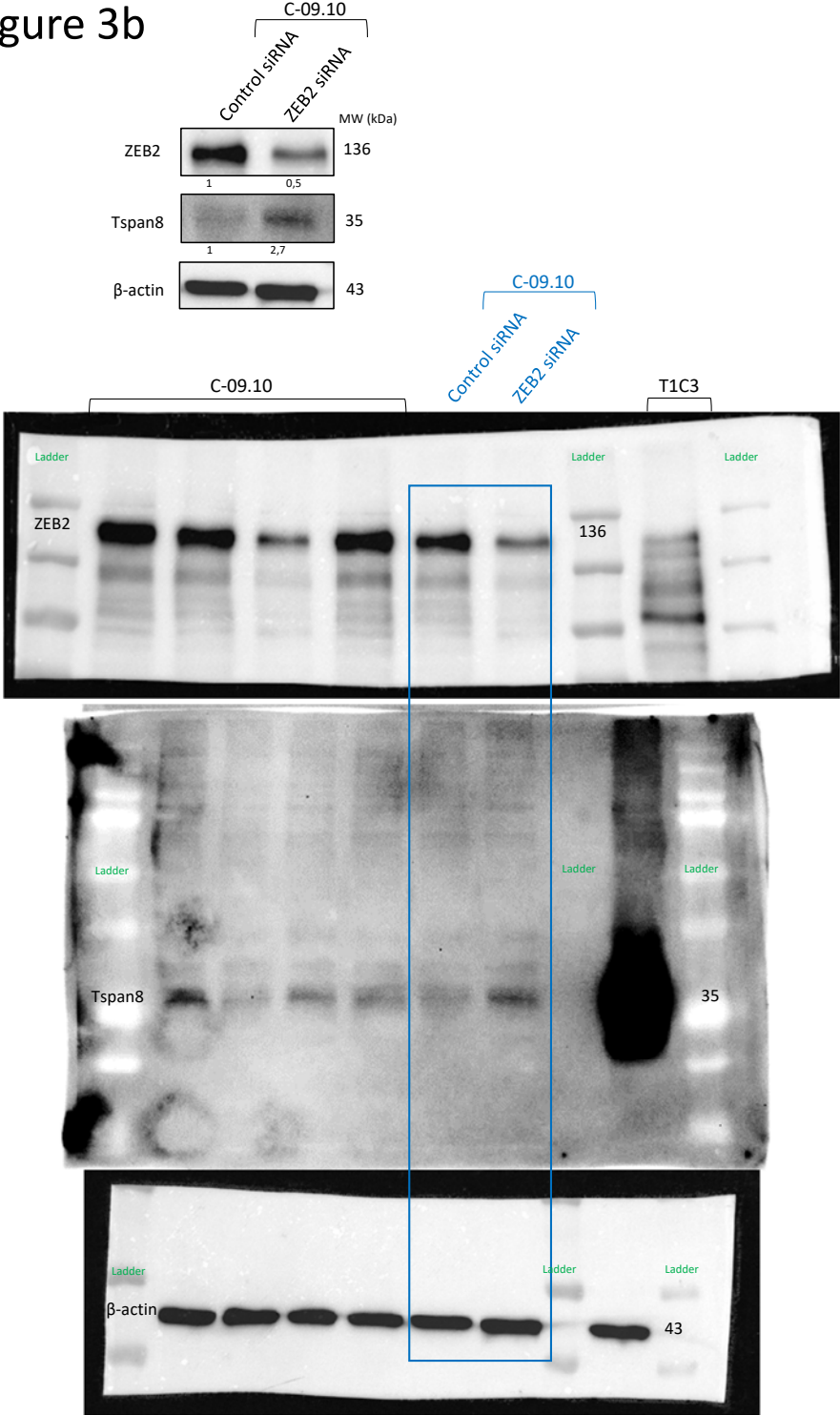

Figure 3c

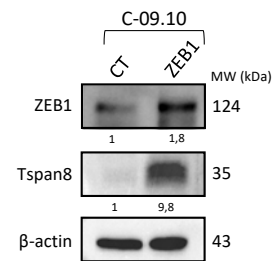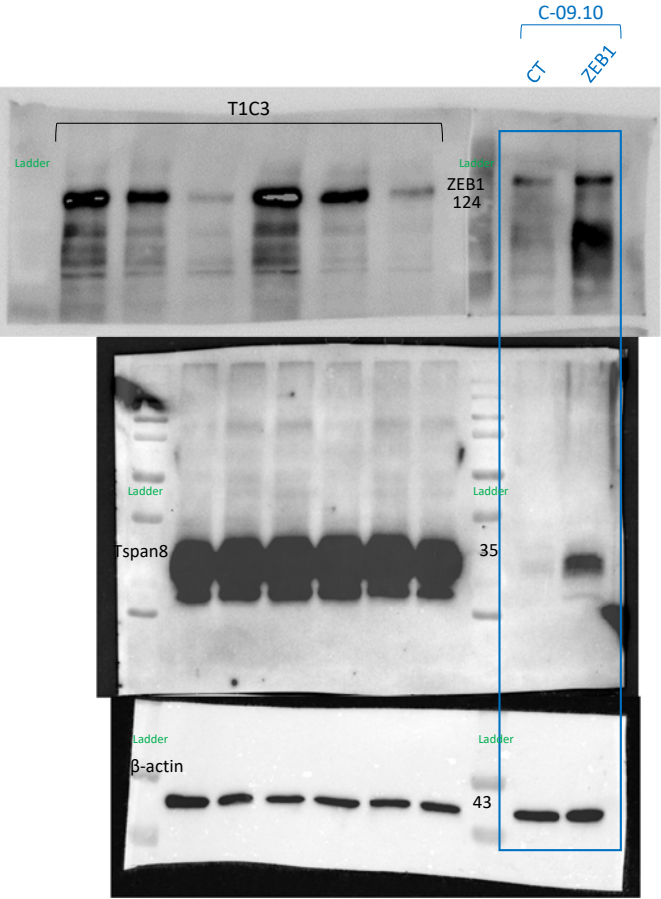

Figure 3e

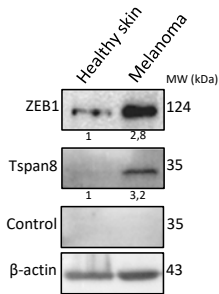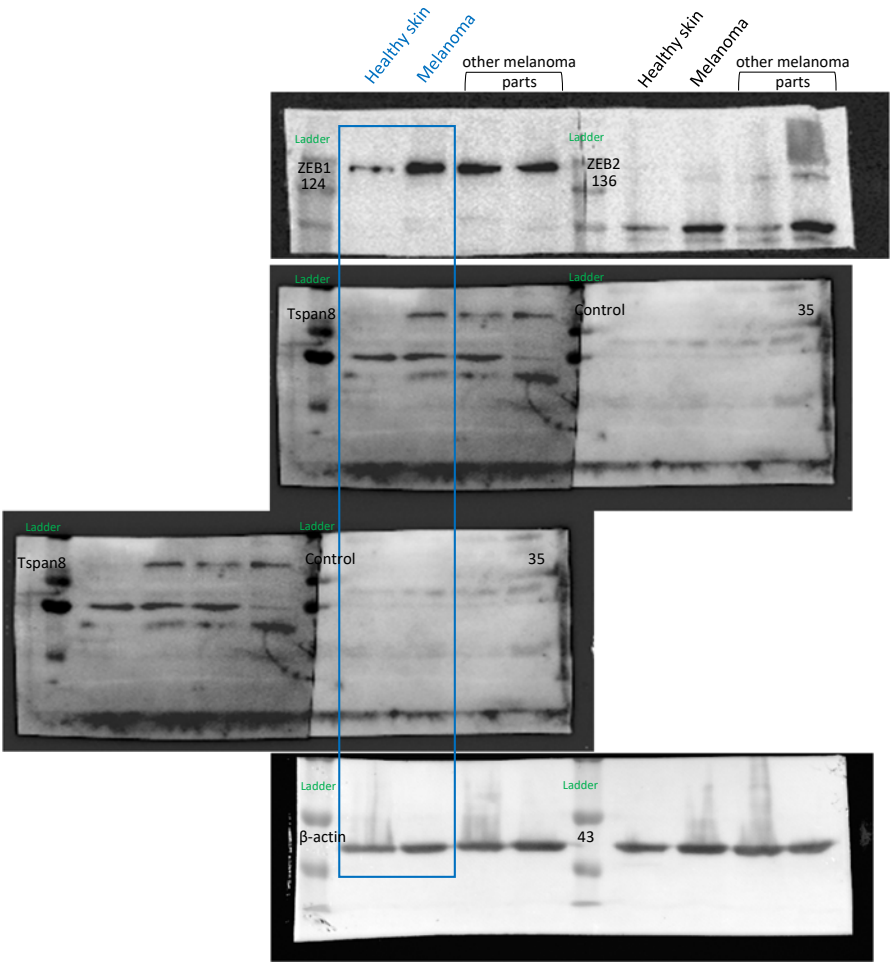

Figure 4a

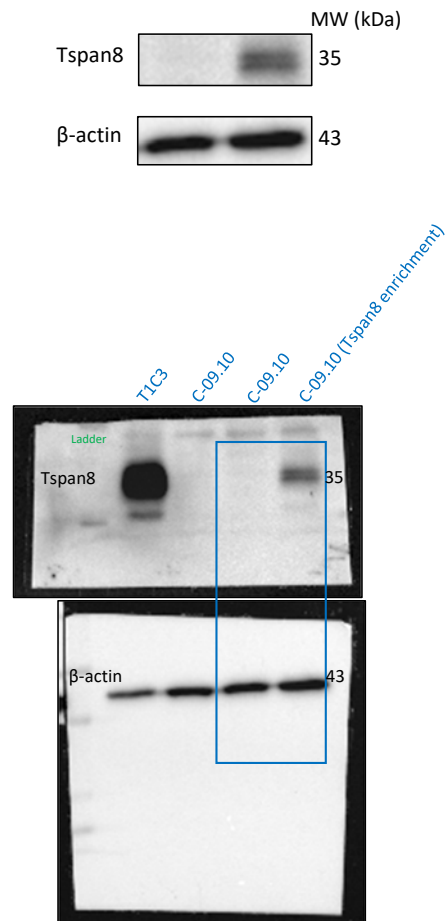

Supplement: Supplementary file 1 [file cancers-16-00694-s001.zip › File S1. The original western blot figures.pdf]
